# Tick–host associations across contrasting habitats in tropical Peninsular Malaysia

**DOI:** 10.1186/s13071-026-07260-0

**Published:** 2026-01-31

**Authors:** Nurul Aini Husin, Muhammad Haiqal Syarriman AbdulRahim, Muhammad Rasul Abdullah Halim, Auni Atikah AbdulHalim, Muhammad Al Amin Mohd-Redzuan, Siti Nur Athirah Azman, Tharane Ganasen, Norhidayu Sahimin, Van Lun Low, Edley A. Jiliun, Ahmad Khusaini Mohd Kharip Shah, Benjamin L. Makepeace, Sazaly AbuBakar, Zubaidah Ya’cob

**Affiliations:** 1 Higher Institution Centre of Excellence, Tropical Infectious Diseases Research and Education Centre (TIDREC), Universiti Malaya, 50603 Kuala Lumpur, Malaysia; 2 Institute for Advanced Studies, Universiti Malaya, 50603 Kuala Lumpur, Malaysia; 3 Department of Wildlife and National Parks, Peninsular Malaysia, KM10, Jalan Cheras, 56100 Kuala Lumpur, Malaysia; 4 Institute of Infection, Veterinary and Ecological Sciences, University of Liverpool, Liverpool, L3 5RF UK; 5 Institute of Biological Sciences, Faculty of Science, Universiti Malaya, 50603 Kuala Lumpur, Malaysia

**Keywords:** Hard ticks, Host–parasite interactions, Habitat specialization, Network analysis, Zoonotic disease, Southeast Asia, One Health

## Abstract

**Background:**

Ixodid ticks are critical vectors of pathogens affecting human, livestock, and wildlife health. In tropical regions, landscape heterogeneity is a key driver of tick–host associations, yet comprehensive studies across diverse habitats remain limited.

**Methods:**

This study investigated tick infestations on a wide range of animal hosts across four major habitat types comprising natural forests, oil palm plantations, rural villages, and urban areas in Peninsular Malaysia from 2022 to 2023.

**Results:**

Of 1277 hosts of 38 families and 79 species examined, 270 (21.1%) were infested with 1985 ixodid ticks, representing 16 ixodid species. The most abundant tick species were *Haemaphysalis wellingtoni* (44.7%), *Amblyomma cordiferum* (19.7%), and *H*. *semermis* (9.6%). Network and correspondence analyses revealed distinct tick–host–habitat associations: *A. cordiferum*, *H. semermis*, *H*. *hystricis*, and *Ixodes granulatus* were strongly associated with natural forests, whereas *H. wellingtoni* predominated in oil palm plantations and rural villages on domestic and jungle fowl (*Gallus gallus domesticus* and *Gallus gallus*). Wild boar (*Sus scrofa*) hosted the most diverse tick species, particularly in urban and rural settings. Notably, *A*. *varanense* exhibited strict specificity to reptiles.

**Conclusions:**

These findings demonstrate the influence of habitat on tick–host interactions, offering critical insights for targeted surveillance and integrated One Health strategies to mitigate tick-borne disease risks in rapidly changing tropical ecosystems.

**Graphical abstract:**

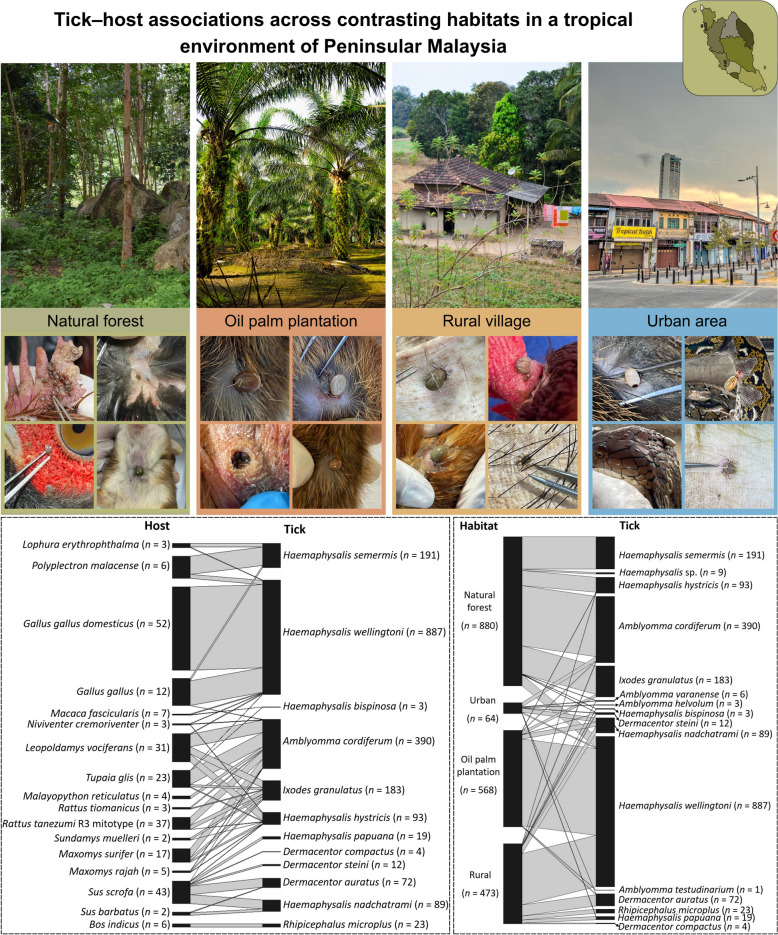

**Supplementary Information:**

The online version contains supplementary material available at 10.1186/s13071-026-07260-0.

## Background

Ticks represent a growing threat to global health, transmitting a greater diversity of pathogenic viruses, bacteria, and protozoa than any other arthropod vector [1, 2]. The escalating incidence of tick-borne diseases (TBDs) such as Lyme disease, anaplasmosis, and spotted fever group rickettsioses is driven by complex environmental changes that alter the interactions between vectors, hosts, and pathogens [1, 3]. In Malaysia, several TBDs, particularly anaplasmosis and spotted fever group rickettsioses, have been documented in both humans and animals [4]. Nowhere is this dynamic more pronounced than in tropical regions, where high biodiversity, overlapping wildlife–livestock–human interfaces, and rapid land-use change create hotspots for zoonotic emergence [5, 6].

In Southeast Asia, and particularly in Malaysia, deforestation for agriculture and urbanization is transforming landscapes at an unprecedented rate [7, 8]. These modifications fundamentally reshape ecological communities, influencing the abundance, diversity, and host-seeking behavior of tick species [9, 10]. While ticks and TBDs are well-studied in temperate systems, the drivers of tick population dynamics and host associations in hyperdiverse tropical landscapes remain poorly understood [11]. Existing studies in Malaysia have provided valuable insights but are often fragmentary, focusing on specific host groups (e.g., small mammals) or isolated habitats, leaving a critical gap in our understanding of tick ecology across entire environmental gradients [12, 13]. A holistic, One Health approach is essential to predict and mitigate TBD risks in changing environments. This requires characterizing the networks of interactions between tick species and their wildlife and domestic hosts across the spectrum of human-modified habitats, from intact forests to urban centers [14, 15].

To address this knowledge gap, we conducted a comprehensive survey of tick infestations on diverse vertebrate hosts, including small and large mammals, birds, and reptiles, across four major habitat types in Peninsular Malaysia: natural forests, oil palm plantations, rural villages, and urban areas. The objectives were to: (1) quantify tick occurrence across habitats; (2) identify key tick–host associations using bipartite network analysis; and (3) assess habitat specificity among tick species. These findings provide critical baseline data on tropical tick ecology and support targeted surveillance and integrated One Health strategies for managing tick-borne diseases in rapidly transforming landscapes.

## Methods

### Research approvals

This study obtained approvals from the Malaysian Department of Wildlife and National Park (DWNP) (approval letter references: JPHL and TN [IP]: 100-34/1.24 Jld 16 [01]; and JPHL and TN [IP]: 100-34/1.24 Jld 19 [55]) for trapping protected wild animals and from the Universiti Malaya Institutional Animal Care and Use Committee (UM IACUC) (approval code: G8/07052020/09012020-01/R and T/22052023/1303202 3-02/R).

### Sampling site

**Fig. 1:**
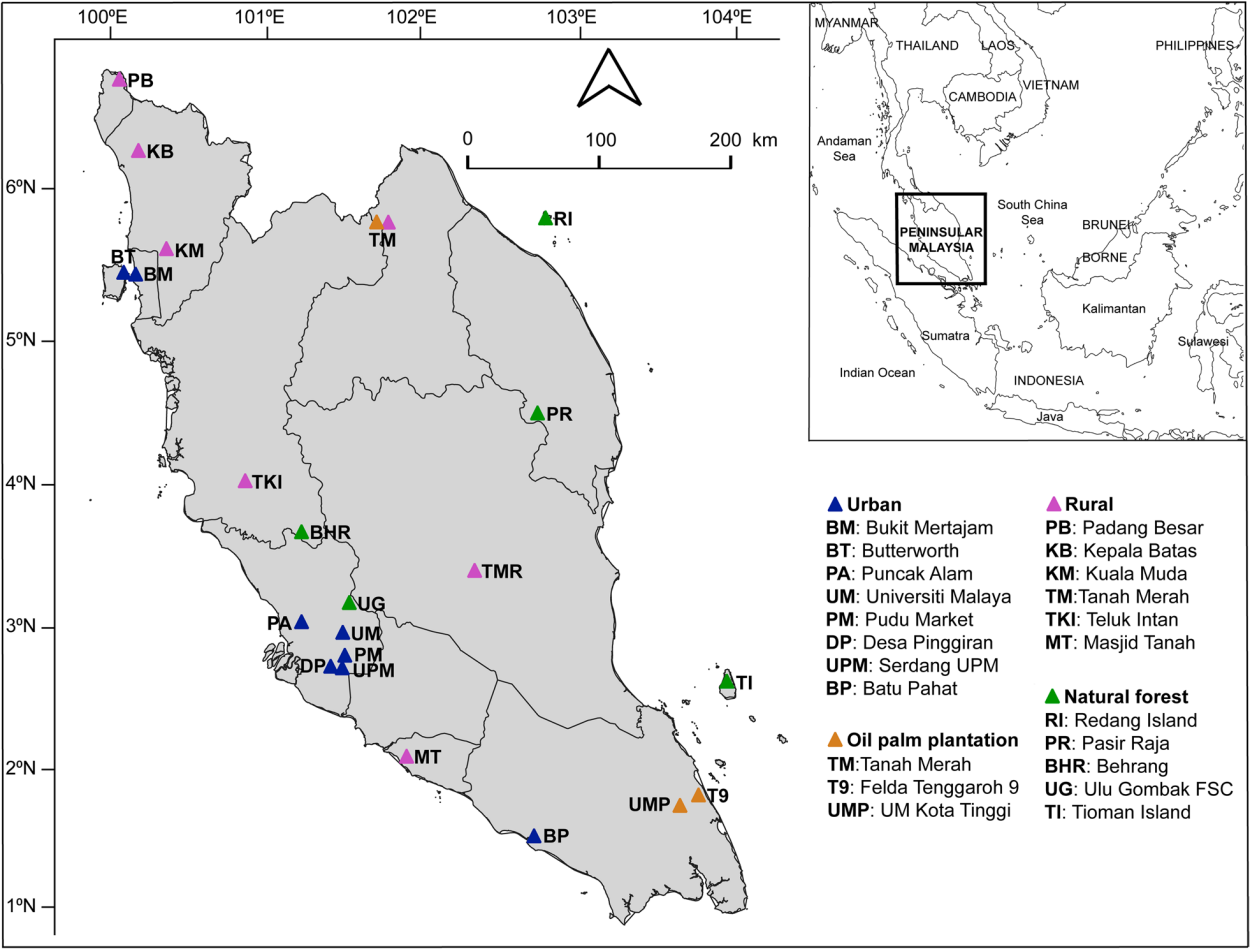
Map of Peninsular Malaysia showing the locations where 22 sampling sites were conducted and the division of four regions: northern, eastern, western, and southern

Figure 1 and Table 1 present the details of the study locations involved in the tick collection from wild and domestic animals in Peninsular Malaysia. On-host ticks were collected from 22 locations across Peninsular Malaysia between May 2022 and October 2023, encompassing four habitats: urban areas, rural villages, oil palm plantations, and natural forests, in four biogeographic regions: the north, south, east, and center of Peninsular Malaysia.

**Table 1:** Tick occurrence on various vertebrate animal hosts across different biogeographic region and habitat types in Peninsular Malaysia between August 2022 and November 2023

State (location)	GPS coordinates	Sampling period	Habitat type	Vertebrate host examined			Tick occurrence	
				Species	Individual (infested)	Infestation rate (%)	Species	Abundance
Johor (UM Kota Tinggi)	N2.02901, E103.86560	July 2023	Oil palm	12	252 (39)	15.5	8	554
Johor (Felda Tenggaroh 9)	N2.09589, E103.98418	Aug. 2023	Oil palm	4	66 (9)	13.6	1	14
Kelantan (Tanah Merah)	N5.75341, E101.97741	Oct. 2023	Oil palm	2	39 (0)	0	0	0
Subtotal (*n* = 3)				12	357 (48)	13.4	8	568
Selangor (Serdang UPM)	N2.98795, E101.72435	Oct. 2022	Urban	2	16 (2)	12.5	3	5
Selangor (Desa Pinggiran)	N2.90751, E101.70826	Oct. 2022	Urban	1	9 (3)	33.3	1	5
Penang (Butterworth & Bukit Mertajam)	N5.42834, E100.30820	Jan.–Feb. 2023	Urban	4	97 (0)	0	0	0
Kuala Lumpur (Pudu Market)	N3.13401, E101.71673	Jan., Oct. 2023	Urban	3	126 (0)	0	0	0
Selangor (Puncak Alam)	N3.20189, E101.44810	Mar. 2023	Urban	1	1 (1)	100	2	2
Kuala Lumpur (UM)	N3.12270, E101.65729	May 2023	Urban	5	13 (1)	7.6	1	3
Johor (Batu Pahat)	N1.83464, E102.93334	July 2023	Urban	4	64 (10)	15.6	6	49
Subtotal (*n* = 7)				11	326 (17)	5.2	8	64
Melaka (Masjid Tanah)	N2.34300, E102.11930	Nov. 2022	Rural village	1	7 (6)	85.7	3	15
Perlis (Padang Besar)	N6.66453, E100.28419	Aug. 2023	Rural village	2	61 (9)	14.7	4	54
Pahang (Temerloh)	N3.52972, E102.55388	Aug. 2023	Rural village	1	6 (6)	100	1	61
Kedah (Kuala Muda)	N5.58260, E100.58486	Sept. 2023	Rural village	2	70 (20)	28.5	6	71
Perak (Teluk Intan)	N4.10010, E101.08821	Sept. 2023	Rural village	1	20 (18)	90	1	164
Kelantan (Tanah Merah)	N5.75341, E101.97741	Oct. 2023	Rural village	2	20 (12)	60	3	85
Kedah (Kepala Batas)	N6.20916, E100.40603	Nov. 2023	Rural village	1	7 (6)	85.7	1	23
Subtotal (*n* = 7)				6	191 (77)	40.3	11	473
Terengganu (Pasir Raja)	N4.53351, E102.95577	Aug. 2022	Natural forest	15	57 (45)	78.9	7	610
Perak (Behrang)	N3.77643, E101.44800	Sept. 2022	Natural forest	36	145 (6)	4.1	4	8
Terengganu (Redang Island)	N5.77889, E103.00692	Oct. 2022	Natural forest	9	88 (38)	43.1	3	135
Pahang (Tioman Island)	N2.82236, E104.16556	May 2023	Natural forest	10	97 (37)	38.1	4	112
Selangor (Ulu Gombak FSC)	N3.32443, E101.75296	June 2023	Natural forest	11	16 (2)	12.5	2	15
Subtotal (*n* = 5)				65	403 (128)	31.7	9	880
Overall				79	1277 (270)	21.1	16	1985

In brief, urban areas were identified with high human population density, marked by concentrated buildings, extensive infrastructure, paved surfaces, limited vegetation cover, and minimal green spaces [16]. In contrast, rural areas were located on the outskirts of urban regions, featuring less developed infrastructure and more abundant vegetation cover [17]. Oil palm plantations consisted of expansive areas dominated by a single cultivated plant species (oil palm), with organized rows of trees and clear boundaries [18]. Natural forests in this study were protected reserve areas under the Malaysia Forestry Department, featuring diverse and intricate ecosystems characterized by a wide variety of tree species, understory vegetation, and wildlife, and are relatively undisturbed or minimally impacted by human activities [19]. All sampling locations (global positioning system [GPS]) were recorded using an Extech RHT3: Ezsmart HygroThermometer device, which includes an integrated GPS module (Extech, NH, USA). Maps were generated from the GPS coordinates using QGIS 3.22.7 software [20].

### Host sampling, tick collection, and identification

#### Small mammals

Small mammals belonging to the families Muridae (rats), Sciuridae (squirrels), Soricidae (shrews), and Tupaiidae (treeshrews) were sampled from all habitats using 100 Hoon’s collapsible traps (41.91 cm × 15.24 cm × 15.24 cm). A variety of baits, including oil palm fruits, dried salted fish, and butterscotch bread, were used [21]. Traps were deployed on the ground and on tree branches along existing trails at ~10-m intervals, set at 1500 h, and checked at 0800 h the following morning. Positive traps were replaced with new ones, while negative traps remained in situ for up to 3 days, with bait replenished daily.

Captured animals were transported to a temporary field laboratory for tick collection. Prior to handling, animals were anesthetized with Zoletil^®^50 via intramuscular injection following protocols approved by the IACUC. Once immobilization was confirmed by the absence of movement and reduced heartbeat, individuals were removed from traps, restrained, and morphologically measured according to Herbreteau and Jittapalapong [22]. Each animal was then examined for ticks across multiple body regions (ears, nose, eyes, upper body, and abdomen). Following examination, animals were returned to cages and released at their capture sites upon full recovery from anesthesia.

#### Ground birds

Ground birds from the family Phasianidae (free-roaming chickens, jungle fowl, and pheasants) were sampled either with owner permission (for chickens, *G. gallus domesticus*) or via trapping during DWNP biodiversity surveys. Chickens were collected at 2000 h using hand nets and housed in temporary steel cages (62 cm × 42 cm × 50 cm) until examination on the following morning (0700 h). Jungle fowl and pheasants were captured using 150 snare traps set in oil palm plantations, rural villages, and natural forest habitats. Each snare was designed to hold one animal at a time. Captured birds were carefully restrained and examined for ticks on the head, comb, belly, thighs, and lower legs. After examination, chickens were returned to their owners, while jungle fowl and pheasants were released at the point of capture.

#### Passeriform birds

Passerine birds were captured using mist nets (12 m × 8 m, 36 mm mesh size) following DWNP protocols. At least 20 nets were deployed daily at fixed locations between 0700 and 1800 h for three consecutive days and inspected every 1–2 h. Nets were closed during adverse weather conditions. Birds entangled in nets were carefully disentangled, placed in individual cloth bags, and examined for ticks for no longer than 10 min per individual. All birds were released at their capture sites following examination.

#### Large mammals

Large mammals from the families Suidae (*S. scrofa*) and Cercopithecidae (*Macaca fascicularis*) were obtained through DWNP’s routine human–wildlife conflict management. Trapping, restraint, euthanasia, and carcass disposal were conducted exclusively by the DWNP. Fresh carcasses were examined for ticks immediately after euthanasia.

#### Reptiles

Reptiles from the families Varanidae (monitor lizards), Pythonidae (pythons), and Elapidae (king cobra) were examined during DWNP biodiversity inventories and through trapping activities conducted in residential areas in response to human–wildlife conflict reports. All trapping, handling, restraint, and species identification were carried out by trained DWNP personnel in accordance with established standard operating procedures. Reptiles were not euthanized for tick collection; all individuals were examined alive. Ticks were carefully removed using appropriate techniques to minimize stress or injury, and upon completion of examination and sample collection, all reptiles were safely relocated to designated natural habitats.

#### Tick collection and identification

All ticks collected from hosts were stored in 2 mL cryotubes (for nymphs and most adults) or 15 mL plastic vials for very large engorged adults (up to 2–3 cm in length), depending on engorgement size. Samples were preserved in liquid nitrogen in the field and subsequently transferred to the −80 °C laboratory at the Tropical Infectious Diseases Research and Education Centre (TIDREC). Tubes were labeled with host code, date, and collection site. Ticks from all developmental stages (larvae, nymphs, and adults) were examined under stereoscopic and compound microscopes and identified to species level using published taxonomic keys [23–25]. Environmental variables, including ambient temperature, relative humidity, and rainfall, were not systematically recorded at all sampling sites. Although portable devices were available during certain field activities, climatic data were collected only at selected locations and time points. As a result, standardized microclimatic measurements across all habitat types were not available and were therefore not included in quantitative analyses relating environmental conditions to tick occurrence.

### Data analyses

Individual host and tick infestation data were compiled into a raw dataset for exploratory ecological analyses. Tick infestation rates and mean tick abundance across different categorical parameters (i.e., host taxa and habitat) were computed using R freeware [26]. The total number of ticks collected from each host individual was recorded and used to calculate descriptive statistics, including the mean and standard error (SE). For each host species, the mean tick burden was computed as the total number of ticks collected divided by the number of hosts examined within that species. Species accumulation curves were generated using the specaccum function in the vegan package in R to visualize the cumulative number of tick species against the total number of host samples examined and to assess the adequacy of sampling effort.

Bipartite network analyses were performed to assess the degree of specialization among tick species concerning their hosts and habitats. These analyses were conducted at individual levels, employing the Bipartite package [27]. Network structure was quantified using several standard network-level indices, including connectance, H2′ specialization, and modularity. These metrics were used to evaluate the overall degree of specialization, interaction redundancy, and compartmentalization within the host–tick network. Additionally, unipartite networks, which illustrate interaction patterns within the host community based on the co-occurrences of tick species, were derived from the bipartite networks using the “tnet” package [28]. Canonical correspondence analysis (CCA) was also utilized to explore the relationship between tick species and habitat types using the “Factoshiny” package.

All ecological analyses were conducted using packages within the R programming environment [26]. As this study was primarily exploratory and descriptive, we focused on visualizing patterns using CCA and bipartite network analyses to highlight habitat-specific tick–host associations.

## Results

### Tick, host, and habitat information

Table 1 presents the geographical details of the study sites, a summary of animal hosts examined at each location, and overall tick infestation and diversity metrics across 22 sites in Peninsular Malaysia, categorized by habitat type and sampling period. In total, 1277 vertebrate hosts representing 79 species were examined from sites distributed across the central, northern, southern, and eastern regions of Peninsular Malaysia, covering natural forests, oil palm plantations, rural villages, and urban habitats. A higher infestation rate was observed in rural villages (40.3%) compared with urban areas (5.2%; Table 1), though sample sizes differed; formal comparison of infestation rates among habitats was not performed.

**Fig. 2:**
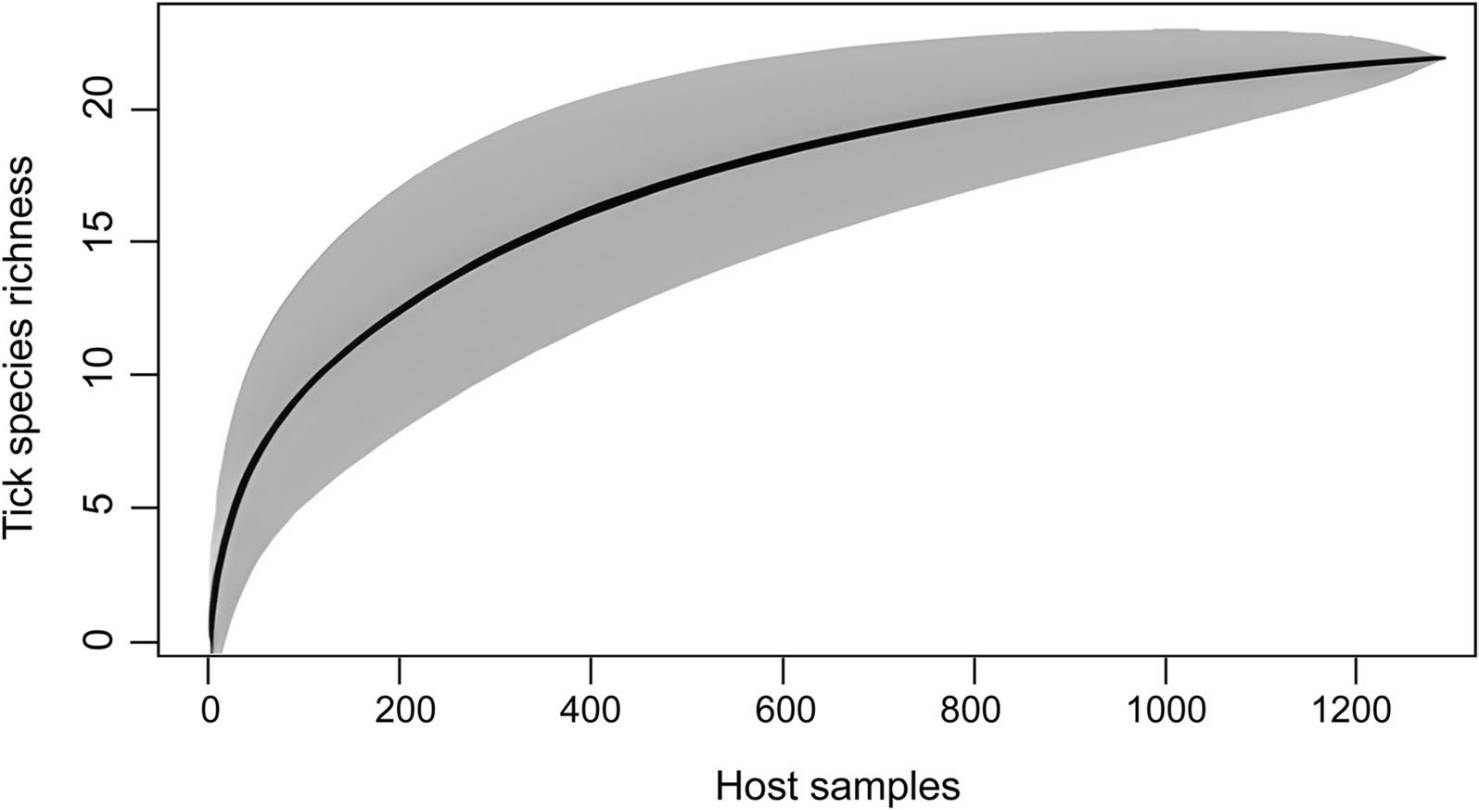
Species accumulation curve for all collections by the four habitat types across Peninsular Malaysia

The host species accumulation curve began to level off as sampling approached 1200 hosts, indicating that most host species likely to harbor ticks in the surveyed habitats had already been captured. This trend suggests sufficient sampling coverage and implies that further host sampling beyond this point would reveal relatively few additional host–tick associations (Fig. 2).

The overall tick infestation rate was 21.1% (270 of 1277 hosts), with variation observed across habitats. Tick burdens per host ranged from 1 to 185 (Supplementary Table S1), with an overall mean of 7.35 ticks per infested host. The highest tick burden in a single host was observed in *G. gallus* (mean 32.3 ± 11.6, range 1–185 ticks), followed by *Polyplectron malacense* (24.4 ± 13.5, range 4–98 ticks) and *Leopoldamys vociferans* (4.2 ± 1.4, range 1–61 ticks) (Table 2 and Supplementary Table S1). As shown in Table 2, tick co-infestations were detected in 15 host species ranging from a minimum of two and a maximum of four tick species with a mean of 1.6 (SE ± 0.2) observed on a single host.

**Table 2:** Details of host species examined and information on their tick infestation in Peninsular Malaysia between August 2022 and November 2023 at four types of ecological habitats, Peninsular Malaysia

Host species	Examined (infested)	Infestation rate (%)	Tick burden			
			Abundance (species richness)	Mean ± SE	Range of co-infestation on single host	
					Min	Max
*Bos indicus*	7 (6)	85.7	23 (1)	3.3 ± 1.3	–	–
*Canis lupus familiaris*	1 (1)	100.0	1 (1)	–	–	–
*Copsychus malabaricus*	25 (1)	4.0	1 (1)	–	–	–
*Crocidura malayana*	1 (1)	100.0	30 (1)	–	–	–
*Gallus gallus*	15 (15)	100.0	485 (2)	32.3 ± 11.6	2	2
*Gallus gallus domesticus*	71 (49)	69.0	362 (1)	5.1 ± 0.8	–	–
*Gonocephalus bellii*	1 (1)	100.0	1 (1)	–	–	–
*Lariscus insignis*	1 (1)	100.0	1 (1)	–	–	–
*Leopoldamys vociferans*	51 (31)	60.8	217 (4)	4.2 ± 1.4	2	4
*Lophura erythrophthalma*	3 (3)	100.0	34 (2)	11.3 ± 0.9	2	2
*Lophura rufa*	1 (1)	100.0	15 (3)	–	3	3
*Macaca fascicularis*	177 (7)	4.0	9 (2)	0.1 ± 0.02	–	–
*Malacopteron magnirostre*	10 (1)	10.0	1 (1)	–	–	–
*Malayopython reticulatus*	4 (4)	100.0	23 (2)	5.8 ± 0.8	2	2
*Maxomys rajah*	7 (5)	71.4	13 (3)	1.9 ± 0.7	–	–
*Maxomys surifer*	25 (17)	68.0	104 (3)	4.2 ± 1.1	2	3
*Maxomys whiteheadi*	7 (1)	14.3	1 (1)	–	–	–
*Niviventor cremoriventer*	6 (3)	50.0	10 (3)	1.7 ± 0.8	2	3
*Ophiophagus bungarus*	1 (1)	100.0	10 (3)	–	3	3
*Paradoxurus musangus*	1 (1)	100.0	1 (1)	–	–	–
*Pellorneum malaccense*	5 (1)	20.0	1 (1)	–	2	2
*Polyplectron malacense*	7 (6)	85.7	171 (2)	24.4 ± 13.5	–	–
*Rattus exulans*	9 (1)	11.1	3 (1)	–	–	–
*Rattus tanezumi* R3 mitotype	280 (37)	13.2	93 (3)	0.3 ± 0.1	2	2
*Rattus tiomanicus*	34 (3)	8.8	13 (2)	0.4 ± 0.3	–	–
*Sundamys muelleri*	2 (2)	100.0	17 (2)	8.5 ± 5.5	2	2
*Sus barbatus*	2 (2)	100.0	25 (3)	12.5 ± 11.5	3	3
*Sus scrofa*	50 (43)	86.0	175 (8)	3.5 ± 0.5	2	3
*Tragulus napu*	1 (1)	100.0	9 (1)	–	–	–
*Tupaia glis*	74 (23)	31.1	133 (4)	1.8 ± 0.8	2	3
*Varanus salvator*	1 (1)	100.0	3 (2)	–	2	2
Others^*^	397 (0)	0.0	0 (0)	–	–	–
Overall	1277 (270)	21.1	1985 (16)	1.6 ± 0.2	2	4

“−” denotes not applicable

^*^The remaining hosts examined showed no tick infestation, with full details provided in the Supplementary Information Table S2

Of the total hosts examined, 270 were infested with 1985 ticks representing 16 species. The predominant species was *H. wellingtoni* (887 ticks or 44.7%). Relatively common species were *A. cordiferum* (390 ticks or 19.7%), but the majority of species were collected at frequencies less than 10% (Table 3). Seven tick species were recorded at very low prevalence (< 1% occurrence on hosts) and exhibited a high degree of host specificity (Table 3). These included *Dermacentor steini*, found on wild boar (*S. scrofa*) and the lizard *Gonocephalus bellii; Haemaphysalis* sp. on the mouse-deer (*Tragulus javanicus*); and *A. helvolum*, *A. varanense*, *D. compactus*, *H. bispinosa*, and *A. testudinarium*, each associated with one, two, or three host species. Some of these rare ticks, such as *A. testudinarium*, are typically associated with larger hosts such as cattle and deer, which were underrepresented in our sampling, likely explaining their low occurrence.

**Table 3:** Details of tick species (*n* = 16) and their relative abundance and infestation rate on 270 vertebrate hosts examined across four habitat types in Peninsular Malaysia between August 2022 and November 2023

Species	Abundance	Relative abundance (%)	Host species (individuals)	Infestation rate (%)
*Haemaphysalis* *wellingtoni* (Nuttall and Warburton, 1908)	887	44.7	10 (81)	6.3
*Haemaphysalis semermis* (Neumann, 1901)	191	9.6	6 (18)	1.4
*Haemaphysalis hystricis* (Supino, 1897)	93	4.7	10 (21)	1.6
*Haemaphysalis nadchatrami* (Hoogstraal, Trapido, and Kohls, 1965)	87	4.4	2 (27)	2.1
*Haemaphysalis papuana* (Thorell, 1883)	21	1.1	1 (5)	0.4
*Haemaphysalis bispinosa* (Neumann, 1897)	3	0.2	2 (2)	0.2
^*^Haemaphysalis sp.	9	0.5	1 (1)	0.1
*Amblyomma cordiferum* (Neumann, 1899)	390	19.7	14 (67)	5.2
*Amblyomma varanense* (Supino, 1897)	6	0.3	3 (3)	0.2
*Amblyomma helvolum* (Koch, 1844)	3	0.2	1 (1)	0.1
*Amblyomma testudinarium* (Koch, 1844)	1	0.1	1 (1)	0.1
*Dermacentor auratus* (Supino, 1897)	72	3.6	2 (23)	1.8
*Dermacentor steini* (Schulze, 1933)	12	0.6	2 (8)	0.6
*Dermacentor compactus* (Neumann, 1901)	4	0.2	1 (3)	0.2
*Ixodes granulatus* (Supino, 1897)	183	9.2	10 (60)	4.7
*Rhipicephalus microplus* (Canestrini, 1888)	23	1.2	1 (6)	0.5
Total	1985		46 (270)	21.1

^*^Haemaphysalis specimens unable to be identified to species level

### Tick–host network analysis and association with habitats

**Fig. 3:**
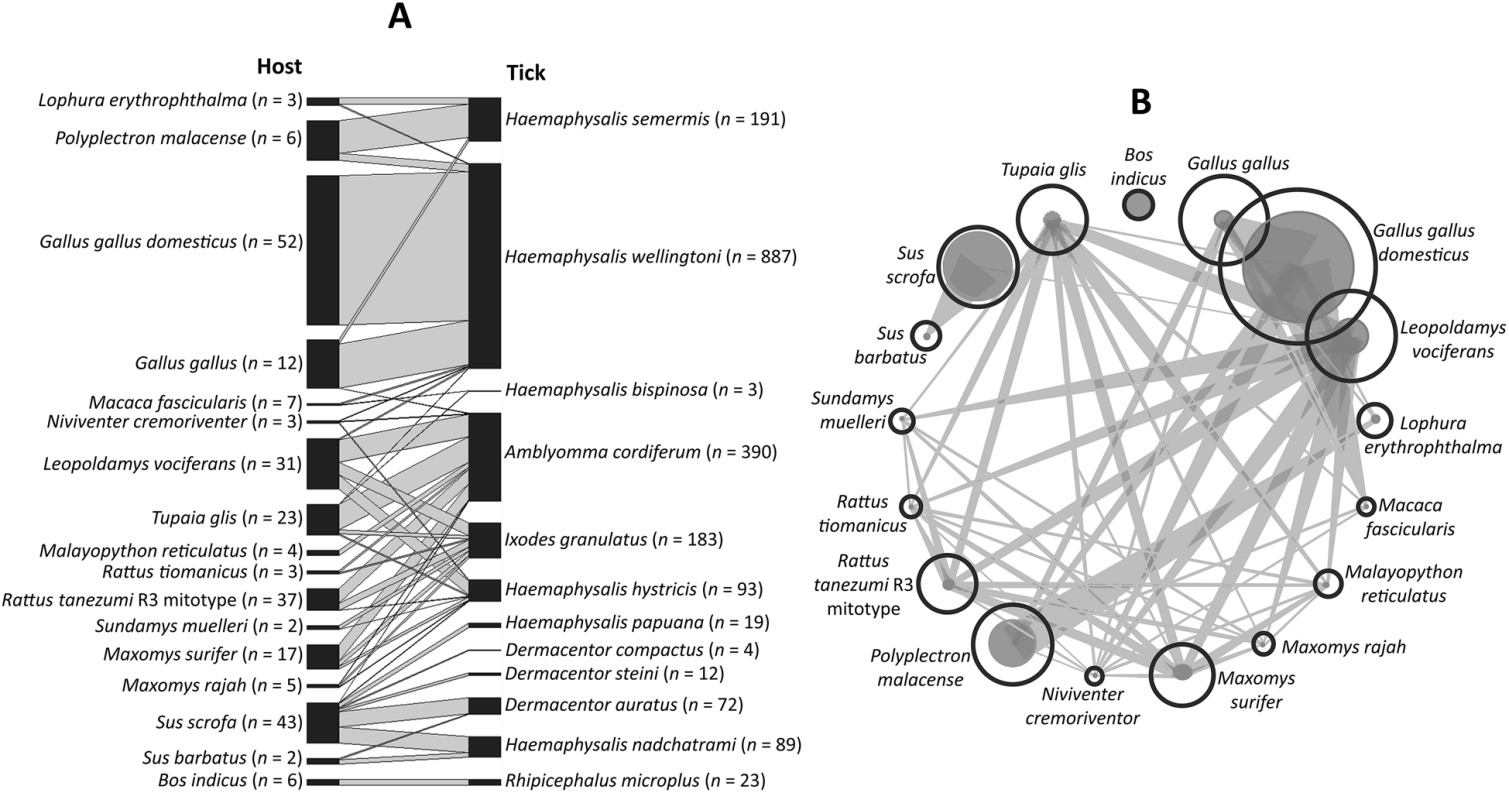
Bipartite network graphs of **A** host–tick associations showing patterns of tick species infestation among different host species and **B** unipartite network of host species linked by shared ticks. Node size shows the number of tick species per host, and node proximity reflects similarity in tick assemblages, highlighting host specialization or generalization

This analysis was conducted after removing 14 host species that were each represented by only a single individual, to avoid biases in the network structure (Fig. 3). As these hosts carried four tick species found only once in the dataset, these tick species were also excluded. The final network comprised 17 hosts (out of 31 infested species) and 12 tick species nodes, revealing heterogeneous host–tick associations, with several tick taxa displaying broad host ranges (Fig. 3A). *Haemaphysalis wellingtoni* and *A. cordiferum* were among the most frequently encountered, parasitizing both domestic and wild hosts, including *G. gallus domesticus*, *G. gallus*, *L. vociferans*, and *T. glis*. *Haemaphysalis hystricis* and *I. granulatus* also demonstrated wide host utilization, infesting small mammals such as *Rattus tiomanicus*, *Maxomys surifer*, and *Sundamys muelleri*. In contrast, several tick species exhibited host specificity. For instance, *Rhipicephalus microplus* was restricted to *Bos indicus* (cattle), while *H. semermis* was found only on avian hosts such as *Lophura erythrophthalma* and *P. malacense*. Similarly, *D. steini*, *D. auratus*, and *D. compactus* were primarily associated with wild boar (*S. scrofa* and *S. barbatus*).

Unipartite network visualization (Fig. 3B) highlighted *G. gallus domesticus*, *S. scrofa*, and *L. vociferans* as central nodes with the highest number of tick associations, indicating their importance as key hosts within the system. Host–tick linkages varied in strength, with thicker edges observed between *G. gallus domesticus* and *H. wellingtoni*, *S. scrofa* and *H. hystricis*, and *L. vociferans* and *I. granulatus*. These associations contributed to a highly connected network, with overlapping interactions observed across multiple host taxa.

Quantitative network metrics supported these qualitative observations. The host–tick interaction network exhibited low-to-moderate connectance (0.225), indicating that only a subset of all possible host–tick interactions occurred. Overall specialization was high (H2′ = 0.628), reflecting nonrandom and selective host use by several tick species. The network was also strongly modular (modularity = 0.732), consistent with the presence of distinct interaction clusters aligned with habitat types and dominant host species.

**Fig. 4:**
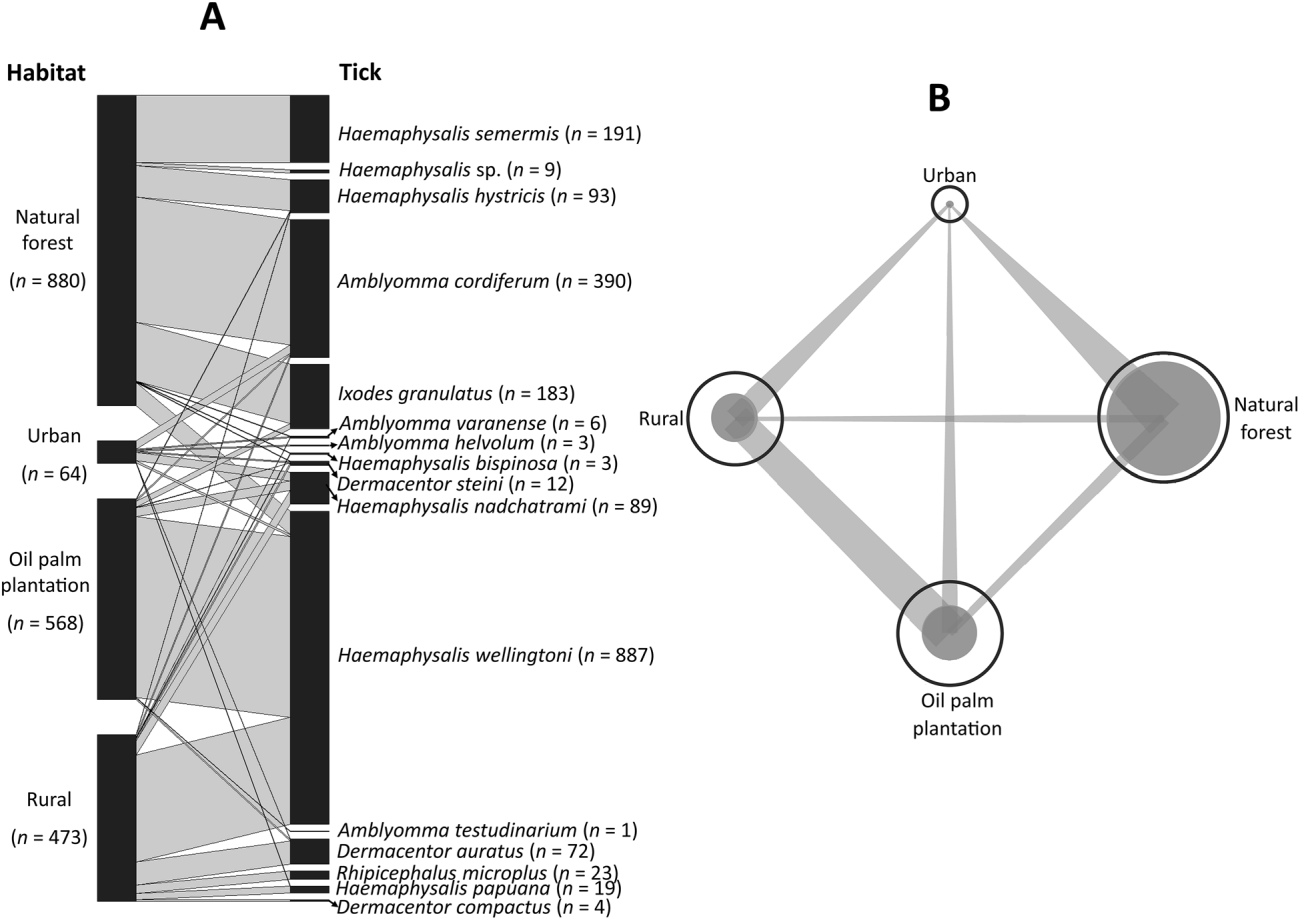
Habitat–tick associations in Peninsular Malaysia. **A** Bipartite network graphs based on presence–absence data. The width of the left bars represents proportional habitat types, and the right bar represents tick abundance, while the linkage width indicates the frequency of interaction. **B** Unipartite network models based on presence–absence data, showing patterns of tick sharing among habitat types

Regarding habitat–tick associations, bipartite network analysis (Fig. 4A) revealed distinct patterns of association between tick species infestation among different habitats where the hosts were captured. Link widths in the network reflect the frequency of tick–habitat interactions, facilitating interpretation of habitat-specific associations. The tick–habitat network exhibited moderate connectance (0.574), low overall specialization (H2′ = 0.217), and low modularity (0.292), suggesting more generalized associations between ticks and habitats with weaker compartmentalization. Several tick species displayed strong habitat specificity. The oil palm plantation and rural village supported the highest number of tick–habitat associations, while urban green areas supported comparatively fewer interactions. Among the recorded species, *H. hystricis* and *A. cordiferum* were strongly linked to forested habitats, with limited overlap across anthropized landscapes. In contrast, *D. steini* and *H. bispinosa* demonstrated broad associations, occurring across both rural village and agricultural habitats. Notably, *I. granulatus* exhibited strict habitat fidelity, with interactions confined exclusively to forest interiors.

Habitat specialization was further reflected in network modularity (Fig. 4B), which partitioned the associations into three distinct clusters (modularity = 0.292; network-level indices): (i) forest-dependent species, (ii) generalist species spanning plantation and rural habitats, and (iii) species confined to rural environments. Interaction strength varied across the network, with the highest weighted links observed between *H. hystricis*–forest and *H. wellingtoni*–plantation (particularly at the village–plantation interface).

### Canonical correspondence analysis (CCA)

**Fig. 5:**
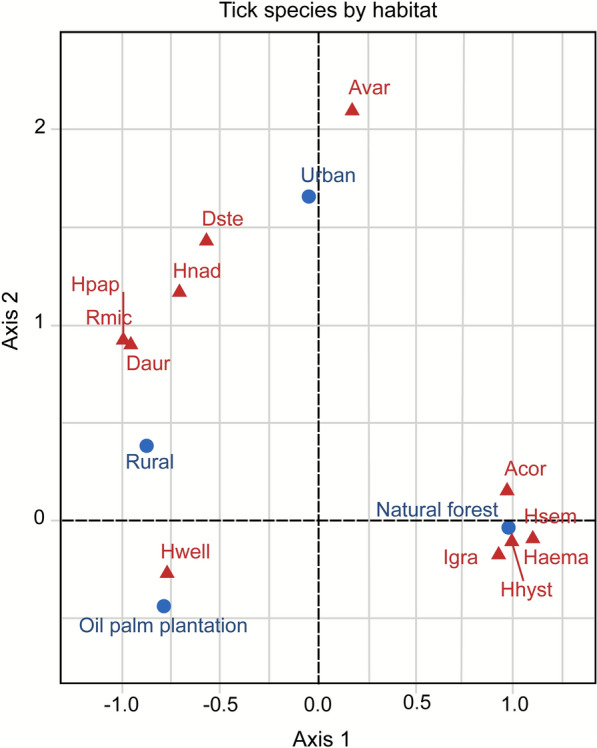
Canonical correspondence analysis showing the associations between the 12 tick species within the four habitat types, with total dimensions explaining 90.08% of total variance. *Amblyomma cordiferum* (Acor), *Amblyomma varanense* (Avar), *Dermacentor auratus* (Daur), *Dermacentor steini* (Dste), *Haemaphysalis* sp. (Haema), *Haemaphysalis hystricis* (Hhys), *Haemaphysalis nadchatrami* (Hnad), *Haemaphysalis papuana* (Hpap), *Haemaphysalis semermis* (Hsem), *Haemaphysalis wellingtoni* (Hwell), *Ixodes granulatus* (Igra), and *Rhipicephalus microplus* (Rmic)

CCA (Fig. 5) indicated that tick species may have distinct habitat preferences regarding land use by the host species, with a clear separation between natural forests, agriculture settings (oil palm plantation), rural village, and urban habitats. The ordination plot displays the first two CCA axes, which together explain 90.08% of the variance, highlighting strong habitat-driven structuring of tick assemblages. Natural forest habitats, which exhibited the greatest species diversity and abundance of ticks overall, particularly on hosts such as *L. vociferans*, were associated with certain tick species: *A. cordiferum*, *H. hystricis*, *H. semermis*, *Haemaphysalis* sp., and *I. granulatus*. In contrast, host species occurring within oil palm plantations were predominantly associated with *H. wellingtoni*. Ticks in rural village sites largely overlapped with plantation ticks, reflecting the presence of generalist species such as *H. wellingtoni*, whereas urban sites supported too few ticks for clear clustering. Statistical significance of axes was not formally tested; however, this separation suggests substantial differences in tick assemblages across habitats.

## Discussion

Our study analyzed tick–host associations across a habitat gradient in tropical Peninsular Malaysia on the basis of the examination of 1277 vertebrates from 79 species; a sample size and taxonomic breadth that significantly exceeds previous local studies [12, 29, 30]. Furthermore, our integrated approach sampled across four distinct habitats (natural forests, oil palm plantations, rural villages, and urban areas), enabling a holistic view of tick community dynamics that has been previously lacking. We revealed a complex pattern of tick community structure where generalist species dominate disturbed habitats while specialists persist in forest interiors, with key wildlife and domestic hosts acting as hubs for potential pathogen transmission.

The overwhelming abundance of *H. wellingtoni*, particularly on domestic fowl (*G. gallus domesticus)* and jungle fowl (*G. gallus*) within oil palm plantations and rural villages, underscores its role as a synanthropic generalist. This finding aligns with previous local studies [31] and suggests that agricultural expansion creates ideal conditions for this species by promoting high-density populations of ground-dwelling avian hosts and providing a humid microclimate under the palm canopy [32, 33]. Similarly, the prevalence of *A. cordiferum* and *H. semermis* highlights the importance of forest-dwelling hosts (e.g., *L. vociferans*) in maintaining significant tick populations, even in modified landscapes.

Crucially, our multivariate and network analyses demonstrated clear tick community partitioning along the habitat gradient. Natural forests harbored a distinct assemblage, including *I. granulatus* and *H. hystricis*, which are strongly associated with forest-dwelling rodents [11, 33]. In stark contrast, oil palm plantations and rural villages formed an ecological continuum dominated by *H. wellingtoni*. This segregation suggests that habitat type is a primary driver of tick community composition, likely mediated through host availability and microclimatic conditions [33, 34].

Network analysis identified two critical hosts: the domestic chicken/jungle fowl (*G. gallus domesticus/G. gallus*) and the wild boar (*S. scrofa*). The former acts as a primary reservoir for *H. wellingtoni* in agricultural settings [35], while the latter, owing to its high mobility and ecological plasticity, and the highest tick species richness observed among hosts in this study, serves as a bridge host, carrying diverse tick fauna, including *Haemaphysalis*, *Dermacentor*, and *Amblyomma* species between natural, rural, and urban habitats. This role positions wild boar as a potential key player in the enzootic maintenance and spillover of tick-borne pathogens [36].

Although jungle fowls and wild boars were identified as important hosts for ticks in this study, direct tick control in free-ranging wildlife is logistically challenging and ethically constrained. Therefore, tick management in these species should not rely on routine chemical treatment of wild animals. Instead, an integrated approach focusing on the wildlife–livestock–human interface is recommended. Practical measures include targeted tick control at forest margins, agricultural buffer zones, and plantation landscapes where wildlife, domestic animals, and humans frequently interact. Environmental management strategies, such as vegetation control, reduction of dense ground cover, and improved landscape hygiene in high-contact areas, may reduce suitable microhabitats for tick survival. In addition, strategic acaricide application on domestic animals that act as bridge hosts (e.g., dogs, livestock) may indirectly limit tick spillover from wildlife to humans. In this context, jungle fowls and wild boars should be regarded primarily as ecological sentinels for tick presence and circulation of tick-borne pathogens, providing early warning signals for zoonotic risk rather than serving as direct targets for tick control interventions.

We also documented strict host specificity, such as the exclusive association of *A. varanense* with large reptiles (*Varanus salvator*, *Malayopython reticulatus*) across habitats. This demonstrates that while habitat disturbance favors generalists, it does not necessarily eliminate specialist interactions if their hosts persist [37, 38].

From a public health perspective, the expansion of generalist ticks such as *H. wellingtoni* into agricultural and peridomestic environments increases the risk of human exposure to tick bites. While the vector competence of most Malaysian ticks remains unconfirmed, members of the genera *Haemaphysalis* and *Amblyomma* are known vectors of serious human pathogens elsewhere, including severe fever with thrombocytopenia syndrome virus (SFTSV) and spotted fever group rickettsiae [39–42]. While the former pathogen has not been recorded in Malaysia, the latter (spotted fever group rickettsioses) have been shown to be important causes of febrile illness in both Peninsular and East Malaysia [43, 44]. The influx of highly mobile hosts such as wild boar into urban fringes further amplifies this risk by potentially introducing ticks and their associated pathogens into human settlements [36, 45].

Several limitations should be considered when interpreting the findings of this study. First, sample sizes were uneven across host species and habitat types, which may reduce the robustness of inferences for rarely sampled hosts. To minimize analytical bias, host species represented by a single individual (*n* = 14) were excluded from the network analysis. In addition, tick collection was restricted to host-associated sampling; consequently, tick species that infrequently parasitize the examined hosts or primarily occur as questing stages in the environment may have been overlooked, potentially resulting in an underrepresentation of the local tick fauna. The absence of argasid ticks is likely attributable to their nidicolous behavior and brief feeding periods, which reduce the probability of detection through host-based sampling without targeted investigation of nests or burrows [46].

Another important constraint is that the vector competence of most tick species in Malaysia remains poorly characterized. As such, interpretations related to tick-borne disease risk are necessarily inferential and based on the documented vector roles of these genera in other geographical regions, rather than on confirmed local transmission cycles. Furthermore, although environmental temperature, humidity, and rainfall are well-recognized drivers of tick survival, activity, and distribution, standardized microclimatic data were not available across all sampling sites. This limitation restricts our ability to assess habitat-specific climatic gradients and to directly evaluate the influence of microclimate on tick occurrence. Accordingly, the observed patterns should be interpreted primarily in terms of host availability and habitat association, rather than as direct responses to environmental or climatic factors.

Despite the abovementioned limitations, this study offers foundational baseline data on tick–host associations across a range of habitat types in Peninsular Malaysia. Future research should aim for more balanced sampling across host species and habitats, incorporate targeted environmental collection of questing and nidicolous ticks, and implement standardized, continuous microclimatic monitoring, including temperature, relative humidity, and rainfall across all sites. Integrating these ecological data with pathogen screening and experimental assessments of vector competence would enhance the rigor of ecological risk assessments and improve predictive models of tick-borne disease dynamics under ongoing land-use change and environmental transformation.

The findings of this study indicate that land-use change in Peninsular Malaysia is linked to shifts in tick community composition, favoring generalist species associated with domestic animals and highly adaptable wildlife hosts. By elucidating tick–host–habitat relationships, the study identifies areas and host species of potential importance for targeted surveillance of tick-borne pathogens. Collectively, these insights advance our ecological understanding of tick dynamics and provide a scientific basis for the development of integrated One Health surveillance and risk assessment strategies for tick-borne diseases in tropical landscapes.

## Conclusions

This study extends beyond species inventories by characterizing patterns of tick–host associations across a heterogeneous landscape. Oil palm plantation environments were associated with a higher occurrence of the generalist tick *H. wellingtoni* on jungle fowl (*G. gallus*) and domestic chickens (*G. gallus domesticus*). In addition, the wide-ranging behavior of wild boar (*S. scrofa*) suggests a potential role as a bridging host, facilitating tick movement between forest remnants and adjacent village or plantation margins.

While this study documents tick occurrence across multiple habitat types in Malaysia on the basis of host examination, the absence of standardized, site-specific climatic data limits the assessment of environmental temperature and other microclimatic influences on tick distribution. Accordingly, the results should be interpreted primarily in terms of host- and habitat-associated patterns rather than as evidence of climate-driven dynamics.

## Supplementary Information

Additional file 1: Table S1. Tick species composition and abundance per host individual, with associated sampling metadata (date, location, GPS, region, and habitat).

Additional file 2: Table S2. Comprehensive details of sampling locations, examined animal species and collected tick species by life stage and sex.

## Data Availability

Data supporting the main conclusions of this study are included in the manuscript.

## Abbreviations

TBDs: Tick-borne diseases
DWNP: Wildlife and National Parks Department
IACUC: Institutional Animal Care and Use Committee
GPS: Global positioning system
TIDREC: Tropical Infectious Diseases Research and Education Centre
SE: Standard error
CCA: Canonical correspondence analysis
SFTSV: Severe fever with thrombocytopenia syndrome virus
